# A predictive model for optimal continuous positive airway pressure in the treatment of pure moderate to severe obstructive sleep apnea in China

**DOI:** 10.1186/s12890-022-02025-8

**Published:** 2022-06-16

**Authors:** Le Wang, Xing Chen, Dong-hui Wei, Mao-li Liang, Yan Wang, Bao-yuan Chen, Jing Zhang, Jie Cao

**Affiliations:** Department of Respiratory and Critical Care Medicine, Tianjin Medical University General Hospital, 154 Anshan Road, Heping District, Tianjin, 300052 China

**Keywords:** Obstructive sleep apnea, Continuous positive airway pressure, Predictive model

## Abstract

**Background:**

Numerous predictive formulas based on different ethnics have been developed to determine continuous positive airway pressure (CPAP) for patients with obstructive sleep apnea (OSA) without laboratory-based manual titrations. However, few studies have focused on patients with OSA in China. Therefore, this study aimed to develop a predictive equation for determining the optimal value of CPAP for patients with OSA in China.

**Methods:**

526 pure moderate to severe OSA patients with attended CPAP titrations during overnight polysomnogram were spited into either formula derivation (419 patients) or validation (107 patients) group according to the treatment time. Predictive model was created in the derivation group, and the accuracy of the model was tested in the validation group.

**Results:**

Apnea hypopnea index (AHI), body mass index (BMI), longest apnea time (LAT), and minimum percutaneous oxygen saturation (minSpO_2_) were considered as independent predictors of optimal CPAP through correlation analysis and multiple stepwise regression analysis. The best equation to predict the optimal value of CPAP was: CPAPpred = 7.581 + 0.020*AHI + 0.101*BMI + 0.015*LAT-0.028*minSpO_2_ (R^2^ = 27.2%, p < 0.05).The correlation between predictive CPAP and laboratory-determined manual optimal CPAP was significant in the validation group (r = 0.706, p = 0.000). And the pressure determined by the predictive formula did not significantly differ from the manually titrated pressure in the validation cohort (10 ± 1 cmH_2_O vs. 11 ± 3 cmH_2_O, p = 0.766).

**Conclusions:**

The predictive formula based on AHI, BMI, LAT, and minSpO_2_ is useful in calculating the effective CPAP for patients with pure moderate to severe OSA in China to some extent.

## Background

Obstructive sleep apnea (OSA) is a kind of common form of sleep-disordered breathing, characterized by cessation or significant attenuation of oronasal airflow during sleep, accompanied by daytime drowsiness and fatigue. It has been reported that nearly one billion adults 30 to 69 years of age worldwide have OSA, and the number of affected individuals is highest in China, followed by the United States, Brazil, and India [1]. OSA has been shown to be associated with multiple-system damage due to intermittent hypoxia [2, 3].

Previous studies have shown that automatic continuous positive airway pressure (auto-CPAP) therapy plays a beneficial role in the treatment of OSA patients [4–6]. However, auto-CPAP titration followed to fixed continuous positive airway pressure (CPAP) under the help of a web platform are not commonly used in China, due to the economic factors and medical insurance policy, as well as the fact that telemedicine services are only at the primary stage with lack of relevant policies and regulations that govern its use at the national level [7]. At present, the main “gold standard” for the diagnosis and treatment of OSA in China includes an initial laboratory diagnostic polysomnogram (PSG), followed by a second PSG to titrate the appropriate value of CPAP manually, if moderate to severe OSA is detected in the baseline PSG [8, 9]. However, the procedure is time-consuming and labor-intensive. Furthermore, the duration of titration may not be sufficient to attain the appropriate pressure because of the patient’s poor ability to sleep in such an unknown environment [10]. In particular, the COVID-19 pandemic has had a major effect on the sleep medicine practices. Laboratory-based sleep diagnosis and treatment of OSA are forced to be postponed or cancelled worldwide, family-based telemedicine is more and more advocated in the current and post-pandemic era [11]. Therefore, it is necessary to find a simple method to determine the CPAP for patients with OSA. Miljeteig and Hoffstein et al. were the first to develop a predictive algorithm to facilitate the selection of initial pressure during an overnight titration study [12]. However, this algorithm may be not suitable for patients with OSA in other countries or regions because of ethnic and regional disparities [13]. Thus, the purpose of this study was to propose a predictive model for determining the optimal value of CPAP for patients with OSA in China.

## Methods

### Study population and design

This study was approved by the Clinical Research Ethics Committee of Tianjin Medical University General Hospital, and all procedures were performed in accordance with the Helsinki Declaration of 1975, as revised in 2000. Written informed consent was obtained from all the participants. We retrospectively evaluated the clinical data of 419 consecutive pure moderate-to-severe OSA patients undergoing CPAP titration at the sleep laboratory of our hospital between March 2016 and August 2019 to determine the predictive model. Thereafter, we validated this predictive formula in a validation cohort of another 107 patients with pure moderate-to-severe OSA between September 2019 and December 2019. We excluded patients who still had high residual AHI (AHI ≥ 5 events per hour) after undergoing CPAP titration, and patients with central sleep apnea/mixed sleep apnea, and patients with severe chronic obstructive pulmonary disease or other severe medical conditions complicated with hypoxia, and patients on sedative/hypnotic medications, and patients with incomplete data.

Demographic information, anthropometric measurements, and medical histories were collected. The neck circumference (NC) was measured at the level of the cricothyroid membrane. The waist circumference (WC) was measured midway between the lower rib margin andanterior superior iliac spine [14].Subjective daytime drowsiness was assessed using the Epworth Sleepiness Scale (ESS), and patients with scores higher than 10 were considered drowsy.

### PSG and CPAP titration for optimal pressure

All patients underwent overnight PSG using a sleep analysis system (Alice 5 diagnostic Sleep System, Philips, Respironics, Bend, OR, USA). Sixteen channels were used simultaneously to perform the following tests: electroencephalogram, electrooculogram, submental and leg electromyogram, electrocardiogram, airflow in the mouth and nose (thermistor, nasal pressure transducer), chest and abdominal respiratory efforts, blood oxygen saturation (pulse oximetry), snoring, and body position parameters. A sleep technician observed the behavior of patients and confirmed their sleep positions through an infrared camera placed in the room [15].

Laboratory-based manual titration was performed throughout the night to determine the optimal pressure for CPAP. Optimal pressure was defined as the lowest effective pressure that could control most respiratory disturbances, including apnea and hypopnea events and snoring in all body positions and stages, especially in the supine position during rapid-eye movement (REM) sleep [15].The specific manifestations were residual AHI < 5 events per hour, and supine REM at least 15 min after treatment [16].

The polysomnographic data were automatically analyzed by the sleep analysis system and were assessed by a polysomnographic technologist. An apnea event was defined as an airflow amplitude reduction of more than 90% from pre-event baseline for at least 10 s, and the hypopnea event was defined as a reduction of airflow by ≥ 30% of pre-event baseline for at least 10 s, and accompanied with ≥ 3% oxygen desaturation from pre-event baseline and/or arousal on electroencephalogram, according to the 2012 American Academy of Sleep Medicine recommendations [17].

### Statistical analysis

The Kolmogorov–Smirnov test was used to assess the normality of distribution. All continuous variables are presented as the mean ± standard deviation (SD) or median with interquartile range (IQR).The Spearman correlation analysis was used to evaluate the relationship between demographic, anthropometric, polysomnographic variables and the observed optimal value of CPAP in derivation cohort. Variables with a significant correlation were considered as potential predictors. Then multiple stepwise linear regression analysis was utilized to select the independent predictive variables and to develop a predictive equation for determining the optimal value of CPAP. In the validation cohort, Spearman correlation analysis was utilized to evaluate the association between optimal CPAP (CPAPopt) and predictive CPAP (CPAPpred) obtained from the predictive formula, and the pressure between the CPAPopt and CPAPpred was compared using the Wilcoxon signed-rank test. Agreement between the CPAPpred and CPAPopt was assessed by the Bland–Altman plot. All statistical analyses were performed using the SPSS version 25.0 (SPSS Inc., Chicago, IL, USA). Statistical significance was set at a two-sided *p* < 0.05.

## Results

### Clinical and polysomnographic characteristics of the study population

The baseline characteristics of patients in the derivation and validation groups were shown in Table 1. The derivation cohort included 419 adult patients with OSA, 73.3% men, age 50.0 (40.0, 59.0) years, BMI 29.4 (27.1, 32.0) kg/m^2^; and a total of 107 adult patients with OSA were included in the validation cohort: 72.9% men, age 49.0 (38.0, 56.0) years, BMI 28.9 (26.9, 30.8) kg/m^2^.

**Table 1:** Demographic, anthropometric, and polysomnographic characteristics of the study population

	Derivation cohort (n = 419)	Validation cohort (n = 107)	p-value
*Demography/Anthropometry*			
Age (year)	50.0 (40.0,59.0)	49.0 (38.0,56.0)	0.212
Gender (male/female)	307/112	78/29	0.938
NC (cm)	42.0 (40.0,45.0)	42.0 (40.0,44.0)	0.110
WC (cm)	106.0 (98.0,113.0)	103.0 (95.0,109.0)	0.003
BMI (kg/m^2^)	29.4 (27.1,32.0)	28.9 (26.9,30.8)	0.131
*Polysomnography*			
ESS (score)	12.0 (6.0,18.0)	8.0 (4.0,14.0)	0.000
AHI (/hr)	57.1 (35.4,71.6)	54.1 (33.2,71.5)	0.659
ODI(/hr)	51.1 (30.2,69.9)	46.8 (25,7,66.9)	0.209
ArI (/hr)	29.1 (15.3,45.3)	25.4 (15.9,44.9)	0.926
AI (/hr)	24.6 (7.3,50.0)	26.4 (9.2,49.3)	0.632
HI (/hr)	21.2 (11.6,34.5)	20.0 (9.9,31.6)	0.137
LAT (s)	54.0 (38.0,68.0)	53.0 (36.0,71.0)	0.964
MAT (s)	23.0 (18.8,27.7)	23.0 (18.2,31.0)	0.531
LHT (s)	56.0 (47,69.5)	62.5 (49.0,78.5)	0.012
MHT (s)	24.4 (20.0,29.0)	24.7 (21.1,30.2)	0.477
REM (%)	10.5 (6.0,15.6)	12.1 (7.4,15.8)	0.175
N1 (%)	28.0 (18.8,42.2)	32.1 (22.1,53.0)	0.021
N2 (%)	52.5 (41.5,61.5)	48.0 (34.5,61.8)	0.101
N3 (%)	4.5 (0.4,10.1)	0.5 (0.0,6.4)	0.000
minSpO_2_ (%)	73.0 (61.0,80.0)	74.0 (62.0,82.0)	0.248
meanSpO_2_ (%)	94.0 (91.0,95.0)	94.0 (91.0,95.0)	0.349
T90 (%)	8.7 (1.5,27.2)	7.9 (1.7,28.1)	0.784

NC, neck circumference; WC, waist circumference; BMI, body mass index; ESS, Epworth Sleepiness Score; AHI, apnea hypopnea index; ODI, oxygen desaturation index; ArI, arousal index; AI, apnea index; HI, hypopnea index; LAT, longest apnea time; MAT, mean apnea time;LHT, longest hypopnea time; MHT, mean hypopnea time;MinSpO_2_, minimum percutaneous oxygen saturation; MeanSpO_2_, mean percutaneous oxygen saturation;T90, proportion of cumulative sleep time with SpO_2_ below 90% in total sleep time;

### The correlations between CPAPopt and demographic, anthropometric, and polysomnographic variables in the derivation cohort

The results showed significantly positive correlations between CPAPopt and variables including NC, WC, body mass index (BMI), ESS, apnea hypopnea index (AHI), oxygen desaturation index, arousal index, apnea index, longest apnea time (LAT), mean apnea time, proportion of cumulative sleep time with oxygen saturation below 90% in total sleep time, and N1%. However, significantly negative correlations were noted between CPAPopt and variables including age, rapid-eye movement (REM %), N3%, minimum percutaneous oxygen saturation (minSpO_2_), and mean percutaneous oxygen saturation. These variables were considered as potential independent predictors for CPAP (Table 2).

**Table 2:** Spearman correlation between optimal CPAP level and collected variables

Variables	Coefficient (r)	p-value
*Demography/Anthropometry*		
Age (yr)	− 0.133	0.006
NC (cm)	0.315	0.000
WC (cm)	0.297	0.000
BMI (Kg/m^2^)	0.320	0.000
*Polysomnography*		
ESS (score)	0.237	0.000
AHI (/hr)	0.441	0.000
ODI (/hr)	0.451	0.000
ArI (/hr)	0.197	0.000
AI (/hr)	0.430	0.000
HI (/hr)	− 0.078	0.112
LAT (s)	0.362	0.000
MAT (s)	0.261	0.000
LHT (s)	− 0.014	0.768
MHT (s)	0.046	0.345
REM (%)	− 0.113	0.021
N1 (%)	0.103	0.035
N2 (%)	− 0.028	0.572
N3 (%)	− 0.154	0.002
MinSpO2 (%)	− 0.384	0.000
MeanSpO2 (%)	− 0.442	0.000
T90% (%)	0.400	0.000

NC, neck circumference; WC, waist circumference; BMI, body mass index; ESS, Epworth Sleepiness Score; AHI, apnea hypopnea index; ODI, oxygen desaturation index; ArI, arousal index; AI, apnea index; HI, hypopnea index; LAT, longest apnea time; MAT, mean apnea time; LHT, longest hypopnea time; MHT, mean hypopnea time;MinSpO_2_, minimum percutaneous oxygen saturation; MeanSpO_2_, mean percutaneous oxygen saturation;T90%, proportion of cumulative sleep time with SpO_2_ below 90% in total sleep time

### Derivation of prediction formula for optimal CPAP level

The potential independent predictors, as shown in Table 2, were included in multiple stepwise linear regression analysis. Finally, AHI, BMI, LAT, and minSpO_2_, were identified as independent predictors in the final predictive model. The final predictive formula for CPAP was:

$$CPAPpred = 7.581 + 0.020*AHI + 0.101*BMI + 0.015*LAT - 0.028*\min SpO2$$

This equation accounted for 27.2% of the total variance (R^2^ = 27.2%, *p* < 0.05) (Table 3).

**Table 3:** Multiple stepwise regression analysis to predict optimal CPAP level

Model	R^2^	Predictors	B	β	T	p-value
	0.272	Constant	7.581		5.066	0.000
		AHI	0.020	0.198	3.505	0.001
		BMI	0.101	0.199	4.005	0.000
		LAT	0.015	0.162	2.700	0.007
		minSpO_2_	− 0.028	− 0.165	− 2.421	0.016

AHI, apnea hypopnea index; BMI, body mass index; LAT, longest apnea time; minSpO_2_, minimum percutaneous oxygen saturation

### Validation of the predictive formula in the validation cohort

**Fig. 1:**
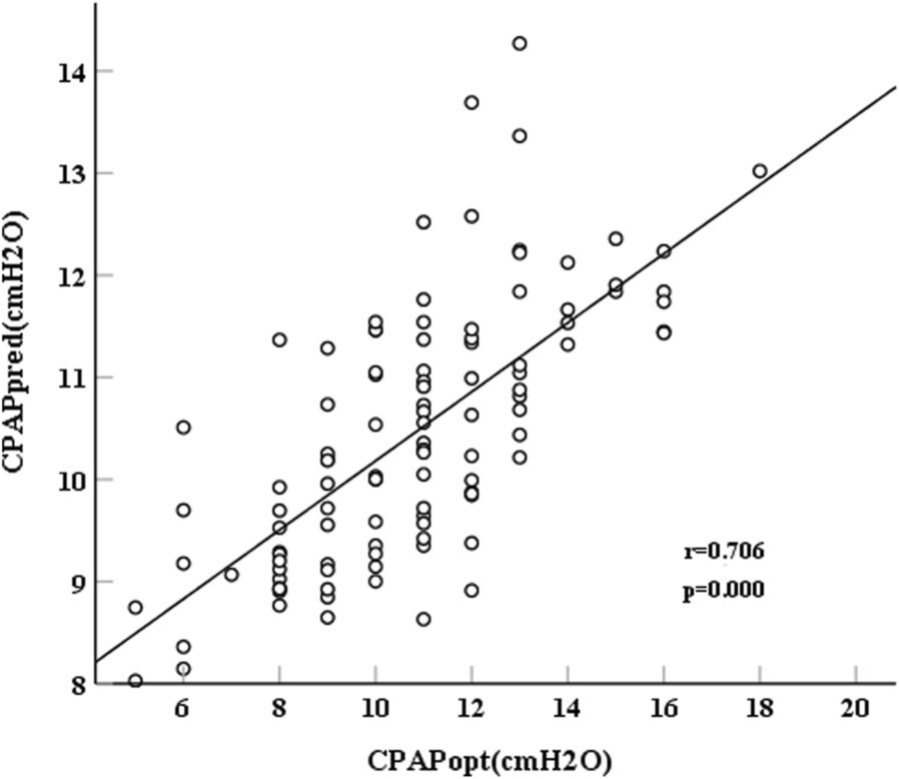
Correlation between CPAPpred and CPAPopt in the validation group

CPAPpred was validated in a separate group of 107 patients with OSA to evaluate the performance of the predictive formula. Just as Fig. 1 showed, a significant correlation was observed between CPAPpred obtained from the predictive formula and CPAPopt obtained by laboratory-based manual titration (r = 0.706, *p* = 0.000).

**Fig. 2:**
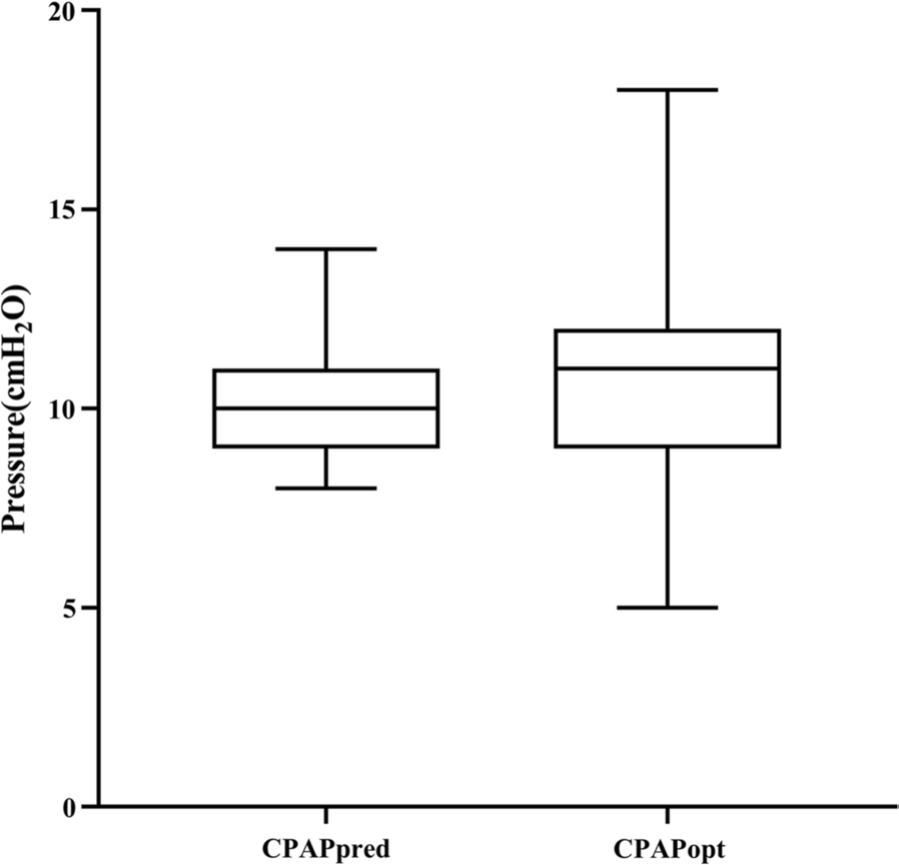
Box-plot of the CPAPpred and CPAPopt in the validation group

In addition to, there was no significant difference between the CPAPopt and the CPAPpred (10 ± 1cmH_2_O vs. 11 ± 3cmH_2_O, *p* = 0.766) (Fig. 2).

**Fig. 3:**
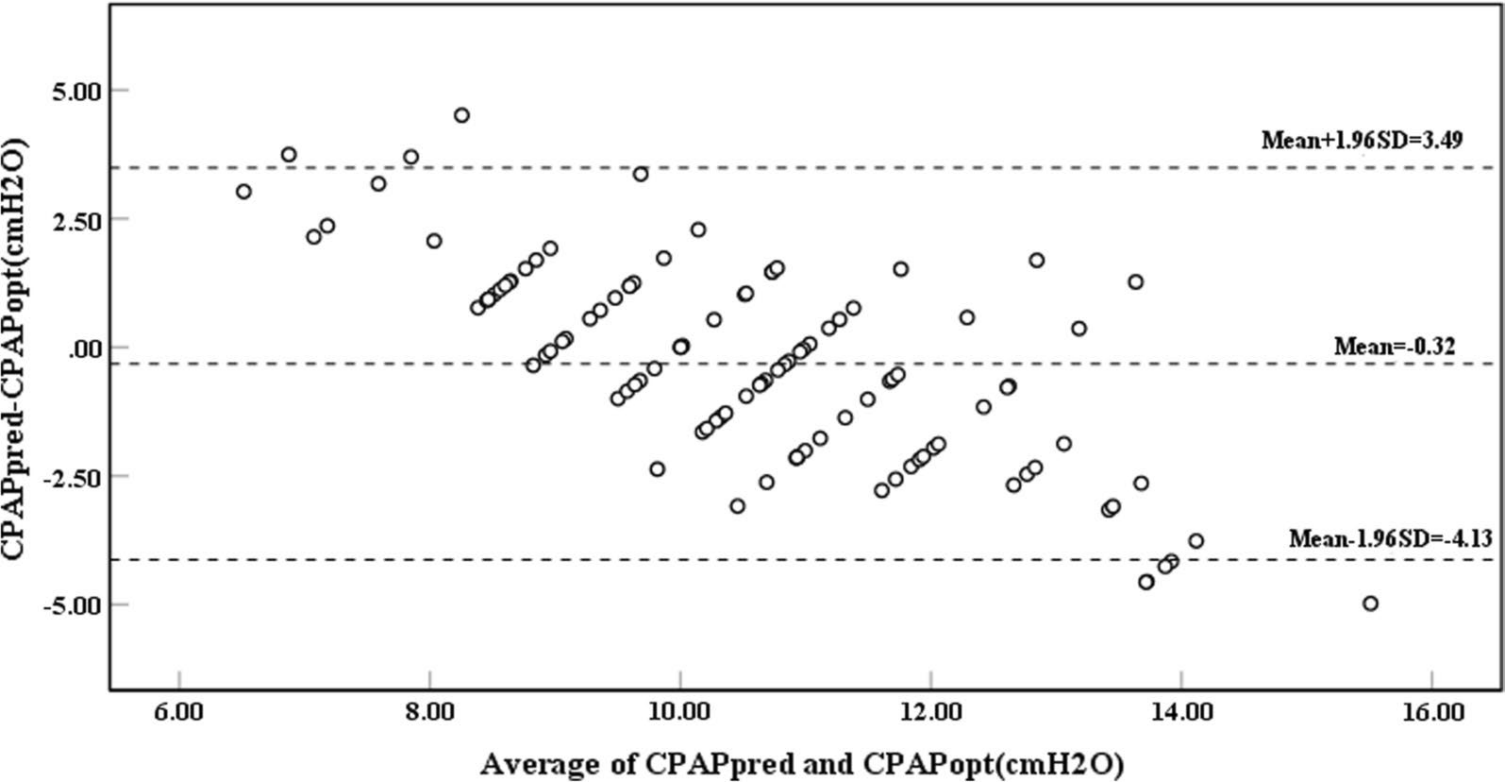
Bland–Altman plot for CPAPopt *vs.*CPAPpred in the validation group

The Bland–Altman plot showed that 5.6% of CPAPpred were without 95% confidence interval (CI) of the calculated mean CPAP difference. In 95% CI, the average value of differential pressure was 0.32cmH_2_O, and the maximum value of that was 4.13 cmH_2_O (Fig. 3).

## Discussion

There is an increasing evidence to support OSA as an independent risk factor for a variety of diseases, such as cardiovascular, cerebrovascular, metabolic, and digestive disorders [2, 3]. The group of disorders have tendency to worsen over time if no or inappropriate treatment is provided. Therefore, early diagnosis and effective treatment in patients with OSA are important [18]. Although various treatment options, including weight control, position therapy, oral appliances, and surgical modifications of the upper airway, have been suggested for the management of OSA, CPAP has been the first-choice of treatment since its introduction in 1981 [19]. However, high level of non-adherence to CPAP treatment is a major limitation of this mode of therapy. Optimal value of CPAP is one of the most important factors influencing adherence, because a lower value of CPAP may result in insufficient treatment and/or unintentional mask removal, whereas a higher value of CPAP may induce pressure intolerance and/or mouth dryness [20]. Thus, achieving the optimal value of CPAP is necessary in patients with OSA. The conventional method of laboratory-based manual titration is time-consuming, labor-intensive, expensive, and delays prescription. Some patients even have to repeat the titration multiple times to get the optimal value of CPAP. Especially, since the outbreak of the COVID-19 epidemic, laboratory-based sleep medicine activities were forced to be postponed or stopped worldwide, and more and more family-based telemedicine have been advocated, which poses a greater challenge to determine the optimal value of CPAP for medical staff. Hence, we proposed a predictive formula for determining the optimal value of CPAP for patients with pure moderate to severe OSA.

The results in this study showed that BMI was one of the important influencing factors of the optimal CPAP. Obesity is widely recognized as a risk factor for OSA, and a pattern of fat distribution around the neck, torso, and abdominal viscera is strongly linked to the pathophysiological mechanism of OSA [21]. Camacho et al. systematically reviewed the international literatures for studying the mathematical equations used to determine the values of effective pressures for CPAP devices. They concluded that BMI were the most important independent predictors of optimal value of CPAP [22]. Extensive clinical researches have shown that BMI was one of the independent factors affecting the optimal CPAP [16, 23–25] (Table 4).

**Table 4:** Mathematical equations to predict CPAP level for patients with OSA in literatures

Study group, year	Country	Number of patients, Development (D)/Validation (V)	Mathematical equation
Ebben MR [8]	USA	Oronasal mask D = 66 V = 66 Nasal mask D = 100 V = 100	Oronasal mask 0.03 × AHI-0.130 × minSpO_2_ + 19.732 Nasal mask 0.017 × AHI-0.092 × minSpO_2_ + 0.225 × NC + 5.534
SaiphoklangN [16]	Thailand	D = 180 V = 0	4.614 + 0.173 × NC + 0.067 × BMI + 0.030 × RDI-0.076 × minSpO_2_
Wu MF [23]	China	D = 57 V = 30	6.380 + 0.033 × AHI-0.068 × minSpO_2_ + 0.171 × BMI
Lee GH [24]	Korea	D = 178 V = 178	6.656 + 0.156 × BMI-0.071 × minSpO_2_ + 0.041 × RDI + 0.094ESS
Schiza SE [25]	Greece	D = 1111 V = 0	Men 5.16 + 0.003 × smoking in pack years + 0.054 × BMI + 0.016 × AHI-0.403 Women 5.16 + 0.003 × smoking in pack years + 0.054 × BMI + 0.016 × AHI-0.806
Liu JH [26]	China	D = 134 V = 0	− 0.7656 + 1.3148 × Sex + 0.2147 × Neck + 0.0175 × LAT + 0.0291 × AHI
Xia SY [27]	China	D = 139 V = 0	2.878 + 0.035 × AHI + 0.035 × LAT + 0.034 × WC
Loredo JS [29]	USA	D = 76 V = 0	30.8 + RDI × 0.03-minSpO_2_ × 0.05-meanSpO_2_ × 0.2

AHI, apnea hypopnea index; minSpO_2_, minimum percutaneous oxygen saturation; NC, neck circumference; BMI, body mass index; RDI, Respiratory disturbance index; ESS, Epworth Sleepiness Score; meanSpO2, mean percutaneous oxygen saturation; LAT, longest apnea time; WC, waist circumference

AHI is the gold standard for the diagnosis and severity classification of OSA. We declared that AHI was significantly correlated with optimal value of CPAP. Consistently, previous studies have demonstrated AHI was the independent factor for determining the optimal value of CPAP [8, 23, 25–27]. Tsuiki et al*.* reported that patients with higher AHI required higher CPAPs to manage OSA [28] (Table 4).

Our results declared that minSpO_2_ was also an independent predictor for optimal value of CPAP. This was similar to several previous studies [16, 23, 24, 29]. Oxyhemoglobin desaturation during sleep is directly related to the duration of apnea and indirectly to end-tidal lung volume at the beginning of apnea, without considering other causes such as lung diseases and/or hypoventilation. Oxyhemoglobin saturation during sleep can be considered as a marker of the severity of OSA, and lower the saturation, greater the CPAP required for its correction [29] (Table 4).

LAT was shown to be another significant contributor to CPAP, which was consistent with those from previous reports. Two studies based on a small sample of patients with OSA in China found that LAT was an independent risk factor for CPAP [26, 27] (Table 4).

In order to verify the accuracy of the formula, we validated the equation in another 107 patients with moderate to severe OSA. We found a significant strong correlation between CPAPopt and CPAPpred. Previous research has found similar correlations. Lee et al*.* found that predictive pressure was positively correlated with titrated pressure (r = 0.490, *p* < 0.001) [24]. Choil et al*.* also reported that pressure determined using their predictive equation was strongly correlated with the full-night titrated pressure (r = 0.883, *p* < 0.001) [19]. In another study conducted in 250 Turkish patients with OSA, the manually measured optimal pressure was significantly correlated with the pressure determined using predictive equation (r = 0.651, *p* < 0.001) [18]. Additionally, the results declared that the pressure of CPAPpred did not significantly differ from that of CPAPopt. Similarly, Choil et al. demonstrated that the pressure determined using full-night manual titration was not different from the pressure determined using the predictive formula (9.0 ± 3.6 cmH_2_O vs.8.1 ± 1.6 cmH_2_O, *p* = 0.080) [19]. Schiza et al. also found that CPAPpred was not statistically different from CPAPopt (7.26 ± 0.5 cmH_2_O vs. 6.44 ± 1.3 cmH_2_O, *p* > 0.05) [25]. The predictive formula proposed by us was helpful to obtain the optimal value of CPAP for patients with pure moderate to severe OSA to some extent, which could help sleep clinicians to have a certain understanding of the approximate range of pressure before the titration, and may further contribute to improving the success rate of lab-based manual titration and auto-CPAP titration through a web platform. Fitzpatrick et al*.* have demonstrated that home self-titration of CPAP using a predictive formula was as effective as laboratory manual titration through a full-night polysomnogram, and showed similar compliance and subjective and objective outcomes [30]. Masa et al. found that titration using a predictive formula was as effective as manual titration in patients with severe OSA, and could lower costs and significantly shorten the waiting list [31]. Rowley et al. reported that the use of a predictive formula increased the success rate of manual CPAP titration from 50 to 68% [32].

There were several limitations in this study. Firstly, our study did not consider the effect of gender. Schiza et al. found that gender was a statistically significant factor affecting CPAP [25]. However, gender was not significant predictor for our CPAP prediction, which may be related to the small sample size of female patients. We will further expand the sample size, especially female patients in the future research. Secondly, cephalometric data have been considered as significant predictive factors of optimal value of CPAP [33]. Craniofacial structures were not considered in the derivation of our predictive formula, because the additional cost of craniofacial examination may diminish the clinical utility of this equation. Moreover, other factors such as life style habits, and the type of mask (oronasal mask/nasal mask) may influence predictive value of CPAP. We will continue this research in the future, and incorporate as more relevant factors as possible to further improve the accuracy of the predictive formula. Thirdly, our validation cohort was relatively small, which was related to the significant reduction in the number of patients undergoing lab-based manual titration since the COVID-2019 outbreak. In the future, a prospective cohort study is also needed to determine its accuracy in clinical practice.

## Conclusions

In this study, we found that the predictive formula based on AHI, BMI, LAT, and minSpO_2_ was useful in calculating the effective CPAP in patients with pure moderate to severe OSA in China to some extent. Larger, fully powered studies are needed to determine its efficacy in the future.

## Data Availability

The datasets generated and analysed during the current study are not publicly available because other study involving this data are in the progress, but are available from the corresponding author on reasonable request.

## Abbreviations

NC: Neck circumference
WC: Waist circumference
BMI: Body mass index
ESS: Epworth sleepiness score
AHI: Apnea hypopnea index
ODI: Oxygen desaturation index
ArI: Arousal index
AI: Apnea index
HI: Hypopnea index
LAT: Longest apnea time
MAT: Mean apnea time
LHT: Longest hypopnea time
MHT: Mean hypopnea time
minSpO_2_: Minimum percutaneous oxygen saturation
meanSpO_2_: Mean percutaneous oxygen saturation
T90: Proportion of cumulative sleep time with SpO_2_ below 90% in total sleep time
CPAPopt: Optimal CPAP
CPAPpred: Predictive CPAP
